# Inhibition of oncogene-induced inflammatory chemokines using a farnesyltransferase inhibitor

**DOI:** 10.1186/1476-9255-5-3

**Published:** 2008-02-27

**Authors:** Katharine C DeGeorge, Brent R DeGeorge, James S Testa, Jay L Rothstein

**Affiliations:** 1 Department of Immunology and Microbiology/Otolaryngology-Head & Neck Surgery, Kimmel Cancer Institute, Thomas Jefferson University, Philadelphia, Pennsylvania, PA 19107, USA; 2 Center for Translational Medicine, Department of Medicine, Thomas Jefferson University, Philadelphia, Pennsylvania, PA 19107, USA; 3 Department of Microbiology and Immunology, Kimmel Cancer Institute, Thomas Jefferson University, Philadelphia, Pennsylvania, PA 19107, USA; 4 Amgen, Inc., Seattle, Washington, USA

## Abstract

**Background:**

Farnesyltransferase inhibitors (FTI) are small molecule agents originally formulated to inhibit the oncogenic functions of Ras. Although subsequent analysis of FTI activity revealed wider effects on other pathways, the drug has been demonstrated to reduce Ras signaling by direct measurements. The purpose of the current study was to determine if FTI could be used to inhibit the inflammatory activities of a known Ras-activating human oncoprotein, RET/PTC3. RET/PTC3 is a fusion oncoprotein expressed in the thyroid epithelium of patients afflicted with thyroid autoimmune disease and/or differentiated thyroid carcinoma. Previous studies have demonstrated that RET/PTC3 signals through Ras and can provoke nuclear translocation of NFκB and the downstream release of pro-inflammatory mediators from thyroid follicular cells *in vitro *and *in vivo*, making it an ideal target for studies using FTI.

**Methods:**

For the studies described here, an *in vitro *assay was developed to measure FTI inhibition of RET/PTC3 pro-inflammatory effects. Rat thyrocytes transfected with RET/PTC3 or vector control cDNA were co-cultured with FTI and examined for inhibition of chemokine expression and secretion measured by RT-PCR and ELISA. Immunoblot analysis was used to confirm the level at which FTI acts on RET/PTC3-expressing cells, and Annexin V/PI staining of cells was used to assess cell death in RET/PTC3-expressing cells co-cultured with FTI.

**Results:**

These analyses revealed significant mRNA and protein inhibition of chemokines *Ccl2 *and *Cxcl1 *with nanomolar doses of FTI. Neither RET/PTC3 protein expression nor apoptosis were affected at any dose of FTI investigated.

**Conclusion:**

These data suggest that FTI may be applied as an effective inhibitor for RET/PTC3-oncogene induced pro-inflammatory mediators.

## Background

Autoimmune diseases affect approximately 1 in 30 Americans [1], and can cause significant morbidity in those affected, not uncommonly leading to death. Although the basis for autoimmune disease in humans remains unknown, the interaction between genetic and environmental factors such as aging, chronic stress, hormones, and pregnancy [2] is thought to play a critical role. Although infection of the target organ has been observed to greatly exacerbate autoimmune disease in experimental models, no viral etiology has been found in human disease [3]. One of the most prevalent autoimmune diseases in the U.S. affects the thyroid organ, with approximately 4 million Americans afflicted by some form of thyroid autoimmune disease. Life-long thyroid hormone replacement therapy is the present "gold standard" treatment for thyroid autoimmune disease, but is difficult to manage: with 12 existing dosages of thyroid hormone, many patients are left with sub-clinical hypothyroidism and lingering symptoms such as fatigue, constipation, depression, and weight gain. Importantly, this therapy does not protect against the development of differentiated thyroid carcinomas which may be associated with thyroid autoimmune disease [4].

Although the cause of thyroid autoimmune disease has yet to be defined, clinically-observed links between autoimmune disease and cancer have been documented for more than half a century [5,6]; [7]. Indeed, one of the most commonly appreciated associations is chronic autoimmune thyroiditis and differentiated thyroid carcinoma. Although no significant increased risk for cancer has been identified in patients with autoimmune thyroid disease, a chromosomal translocation resulting in the formation of the mutant RET/PTC fusion protein links these pathologies [8-11]. Definitive evidence that Hashimoto's thyroiditis is caused or exacerbated by RET/PTC3 is not yet available, although sufficient evidence exists to support a direct role for activated RET kinase in inducing the mediators of inflammation *in vitro *and *in vivo *[12-14]. Accordingly, there exists a molecular genetic abnormality that is common to thyroid epithelial cells in cancer and autoimmune disease even though the actual mechanism of progression for each disease is not yet clear.

The RET/PTC family are fusion proteins that result from a chromosomal rearrangement involving the tyrosine kinase domain of the c-RET proto-oncogene, and are frequently found in the early development of differentiated thyroid carcinomas [15-21]. The fusion oncoprotein RET/PTC3 (also known as RP3, indicating mouse/human gene or protein) is the most frequent isoform that develops in childhood thyroid cancers, and involves the partnering of the c-RET kinase domain with the androgen receptor-related protein RFG/ARA70. RP3 has been shown to signal through the Ras pathway, and results in nuclear localization of NFκB and the production of pro-inflammatory mediators [22]. Based on an array of over 200 genes activated by RP3, two of the most highly induced are the pro-inflammatory chemokines monocyte chemoattractant protein-1 *Mcp1 *(*Ccl2*) and *Kc/Groα *(*Cxcl1*) [23].

Given that molecular changes may be occurring in thyroid tissue at early stages of disease, treatments that might ameliorate the effects of oncogene-induced inflammatory mediator production may reduce the morbidity associated with thyroid inflammation. Presently existing compounds targeting various signal transduction pathways are available and some, like those that target Ras signaling, have already entered the clinic. Small molecule agents such as the farnesyltransferase inhibitors (FTI) show target selectivity in many models [24]. FTIs represent a group of compounds that inhibit the enzymatic properties of farnesyltransferase, an enzyme important for the post-translational lipid modification of membrane associated proteins, including those of the RAS pathway. FTIs were developed to take advantage of the membrane localization requirements held by many of the molecules in the RAS pathway, known to be some of the most commonly mutated genes in human cancer [25]. By blocking the membrane localization of RAS and associated molecules, FTI functions to suppress proliferation and angiogenesis by inhibiting NFκB activation and expression of NFκB-regulated genes induced by carcinogens and inflammatory stimuli [26]. Despite their potential target specificity, low toxicity, and potential for cancer-specific targeting, these compounds have only been marginally successful for the treatment of advanced malignancies in clinical trials. One explanation for these failures may be that FTIs modulate alternate targets and the assumed dependency on Ras signaling in cancer may not hold up in all stages of tumor development. Interestingly, recent studies describe FTIs as anti-inflammatory agents and found significant efficacy in both cell- and animal-based models of inflammation [26,27]. For this reason, we have chosen to use FTI to study the inhibition of RP3-induced inflammatory mediators produced by oncogene-transfected thyroid cells. The extension of these studies provide a therapeutic rationale for using FTI in thyroid autoimmune disease.

## Methods

### Materials

The farnesyltransferase inhibitor tipifarnib (ZARNESTRA^®^), R115777 [(B)-6-[amino(4-chlorophenyl)(1-methy-1H-imidazol-5-yl)-methyl]-4-(3- chlorophenyl)-1-methyl-2(1H)-quinolinone] was supplied by Dr. David End of Johnson & Johnson, Beerse, Belgium. For each experiment, stocks were prepared fresh daily from R115777 powder in DMSO and protected from light.

### Cell culture

PC Cl3 rat thyrocytes previously obtained from Dr. Jeffrey Knauf (University of Cincinnati, Cincinnati, OH, USA) were stably transfected with human RP3 via ligation of RP3 onto the retroviral vector, pMV7 as described previously [23]. The RP3-transfected thyrocytes (PC Cl3^RP3^) were grown in Coon's Modified F12 medium (Sigma, St. Louis, MO, USA) supplemented with 7.5% fetal bovine serum, 2 mM L-glutamine, and 100 U/ml penicillin/streptomycin. PC Cl3 cells containing pMV7 vector only (PC Cl3^pMV7^) were additionally supplemented with the following growth factors: 10 ng/ml somatostatin, 10 ng/ml glycine-histidine-lysine, 5 μg/ml transferrin, 10 nM hydrocortisone, 10 μg/ml insulin, and 10 mIU/ml bovine thyroid stimulating hormone. Cells were grown in a water-saturated environment in 5% CO_2 _and 95% air at 37°C.

### FTI treatment assay

PC Cl3 rat thyrocytes stably transfected with RP3 (PC Cl3^RP3^) and vector control (PC Cl3^pMV7^) at mid-log phase growth were trypsinized and live cells were counted using a trypan blue stain. Cells were plated on a 12-well polystyrene culture dish at a cell density of 2.5 × 10^5 ^cells per well (65 cells/mm^2^) suspended in 2 ml culture medium. Cells were allowed to adhere and stabilize for 18 hours. At that time, culture supernatants were removed, cells were washed using room temperature 1× PBS, and 2 ml of culture medium containing FTI (at clinically-relevant concentrations of 10,000 nM, 1,000 nM, 100 nM, 10 nM, or 1 nM) or 2 ml of control culture medium (0 nM FTI) was added to each well. DMSO effects (the compound diluent) were controlled for: all culture conditions including control maintained a 0.05% DMSO concentration. Cells were allowed to grow in the presence of FTI-containing media for 24 hours.

### RT-PCR analysis

Total cell RNA was extracted using the TRIzol method (Invitrogen, Carlsbad, CA, USA) of RNA isolation. Precipitated DNA was digested with RNAse-free DNAse (Ambion, Austin, TX, USA). Total RNA (5 μg) was denatured and reverse-transcribed using SuperScript™ III Reverse Transcriptase (Invitrogen, Carlsbad, CA, USA) in a reaction mix containing random primers (50 ng/μl), Oligo dT (105 ng/μl), and 0.1 M DTT for 90 minutes at 42°C. Reverse transcription was confirmed, and the presence of genomic DNA excluded, with PCR of cDNA and similarly incubated but non-transcribed RNA using primers for glyceraldehyde-3-phosphate dehydrogenase (*G3pdh*). cDNA was amplified by PCR using primers specific for rat *G3pdh*, rat *Ccl2*, rat *Cxcl1*, and human RP3 (breakpoint region). Amplified products for chemokine-specific primers and h-RP3 were normalized using the amplified product for *G3pdh*. PCR cycling conditions for all reactions were: denaturation at 94°C for 4 min for 1 cycle; 20 cycles of denaturation at 94°C for 30 sec, primer annealing at 60°C for 30 se, and extension at 72°C for 1 min; and final single extension cycle of 72°C for 7 min. The primer sequences were: rat *G3pdh *[sense: 5' AGAACATCATCCCTGCATCC 3'; antisense: 5' GTCCTCAGTGTRAGCCCAGGA 3']; rat *Ccl2 *[sense: 5' CACTCACCTGCTGCTACTCATTCA 3'; antisense: 5' GCTTGAGGTGGTTGTGGAAAAG 3']; rat *Cxcl1 *[sense: 5' GCGGAGAGATGAGAGTCTGG 3'; antisense: 5' GAGACGAGAAGGAGCATTGG 3']; human RP3 (breakpoint region) [sense: 5' CCAGAGCAGAAGTCAGCATTC 3'; antisense: 5' CTCTTTCAGCATCTTCACGGC 3']. The PCR product sizes were as follows: r-*G3pdh*, 227 bp; r-*Ccl2*, 320 bp; r-*Cxcl1*, 215 bp; and h-RP3, 302 bp. PCR products were visualized using gel electrophoresis (2% agarose gels with 0.5 μg/ml of EtBr) and quantified using the BioRad Gel Doc and Quantity One program (BioRad, Hercules, CA, USA).

### ELISA

CCL2 and CXCL1 secretion were measured using an ELISA according to the manufacturer's protocol (Amersham/GE Healthcare Technologies, Waukesha, WI, USA). PC Cl3^pMV7 ^and PC Cl3^RP3 ^thyrocytes at mid-log phase growth were co-cultured with FTI according to described protocol. Culture media were collected and filtered before use in ELISA. All values refer to the average of duplicate samples from triplicate experiments ± s.e.m.

### Western blot analysis

Immunoblotting was performed to assess protein levels of the tyrosine kinase domain of RP3 using Ret (C-19) goat polyclonal IgG (sc-167, Santa Cruz Biotechnology, Inc., Santa Cruz, CA, USA) and phosphorylated-Ret (Tyr 1062)- rabbit IgG (sc-20252-R, Santa Cruz Biotechnology, Inc., Santa Cruz, CA, USA) in PC Cl3^RP3 ^cells following the FTI co-culture assay described herein. Protein content was quantified with the BioRad DC Protein Assay (BioRad Laboratories, Hercules, CA, USA). Following separation of protein samples (50 μg per lane) by 10–20% SDS-PAGE (Invitrogen, Carlsbad, CA, USA), proteins were transferred to nitrocellulose membranes and probed with primary antibodies at 4°C overnight. After staining with a corresponding pair of IRDye 800CW-coupled anti-goat (Rockland Inc., Gilbertsville, PA; 1:10.000) and Alexa Fluor 680 anti-rabbit (Molecular Probes; 1:10.000) secondary antibodies, respectively, proteins were visualized with a LI-COR infrared imager (Odyssey), (LI-COR, Inc., Lincoln, Nebraska USA) and quantitative densitometric analysis was performed applying Odyssey version 1.2 infrared imaging software. Phospho-RET intensities were normalized to total-RET densitometric levels that were not different between groups.

### FACS analysis of early-apoptotic-staining cells

The Annexin V:FITC Apoptosis Detection Kit I (BD Biosciences, San Jose, CA, USA) was used according to manufacturer's instructions to stain PC Cl3^RP3^thyrocytes grown to mid-log phase growth and co-cultured with FTI according to the previously described protocol. Cellular uptake of FITC-conjugated Annexin V and/or propidium iodide was measured using a Coulter XL (Beckman Coulter, Inc., Fullerton, CA, USA) flow cytometer.

### Statistical analysis

Results are presented as the mean ± standard error of the mean (s.e.m.). Data were analyzed using a KS Normality Test and determined to be of normal distribution. A Student's *t*-test was used to determine the significance of each test sample group (n = 3) compared to the control sample group (n = 3) utilizing the program Microsoft Excel. *P*-values < 0.05 were considered statistically significant for these experiments with a 95% confidence interval.

## Results

### RP3 induces pro-inflammatory gene expression in thyrocytes

**Figure 1:**
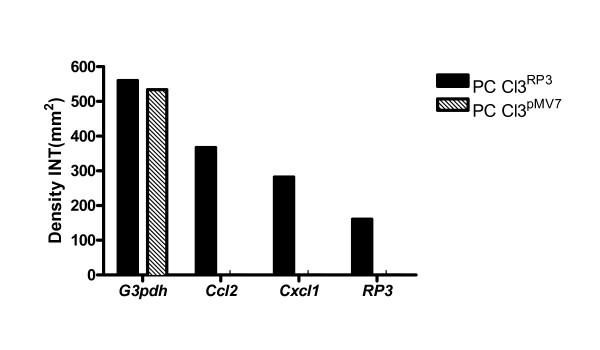
**Oncogene-induced expression of pro-inflammatory genes in RP3-expressing thyrocytes**. Shown is the baseline gene expression of the housekeeping gene *G3pdh*, pro-inflammatory genes *Ccl2 *and *Cxcl1*, and the oncogene RP3 in PC Cl3^RP3 ^and PC CL3^pMV7 ^stably-transfected thyrocytes. Bars depict densitometric analysis of RT-PCR data. Cells expressing RP3 also express the pro-inflammatory genes *Ccl2 *and *Cxcl1*, whereas vector controls do not.

Previous work has demonstrated the induction of pro-inflammatory proteins by the RP3 oncogene [23]. To measure the effects of FTI on these pro-inflammatory mediators, we chose *Ccl2 (Mcp1) *and *Cxcl1 *(*Kc/Groα*) that were expressed at high levels in RP3 transfectants. Baseline expression of the housekeeping gene *G3pdh*, the pro-inflammatory genes *Ccl2 *and *Cxcl1*, as well as the oncogene RP3 was determined using RT-PCR. The choice of chemokines for these experiments was based on previous data [23] which demonstrated that of over 200 differentially expressed genes, *Ccl2 *and *Cxcl1 *(*Kc/Groα*) were two of the top four most highly expressed genes in RP3-expressing thyrocytes when compared to parental cells (vector controls). Figure 1 demonstrates the gene expression of *G3pdh*, *Ccl2*, *Cxcl1*, and RP3 in PC Cl3^RP3 ^and PC Cl3^pMV7 ^cells. There is no expression of RP3, *Ccl2*, or *Cxcl1 *by the vector control thyrocytes (PC Cl3^pMV7^), data which is consistent with the lack of inflammation from these cells. Expression of RP3 directly correlated with production of inflammatory genes as previously described [23,28].

### FTI inhibits pro-inflammatory gene expression in RP3-expressing thyrocytes

Increasing evidence indicates that cancer at its earliest stages is dependent on inflammation [29-31]. Although the cause of this cancer-associated inflammation is currently under investigation, several reports [32,33] indicate that oncogene signaling can be directly responsible for the production of inflammatory mediators. Here, we investigated the idea that inhibition of oncogene signaling may alleviate the downstream production of selected mediators. Previous experiments determined that expression of *Cxcl1 *and *Ccl2 *were correlated with the expression of active, but not signaling-deficient forms of, RP3 [23].

**Figure 2:**
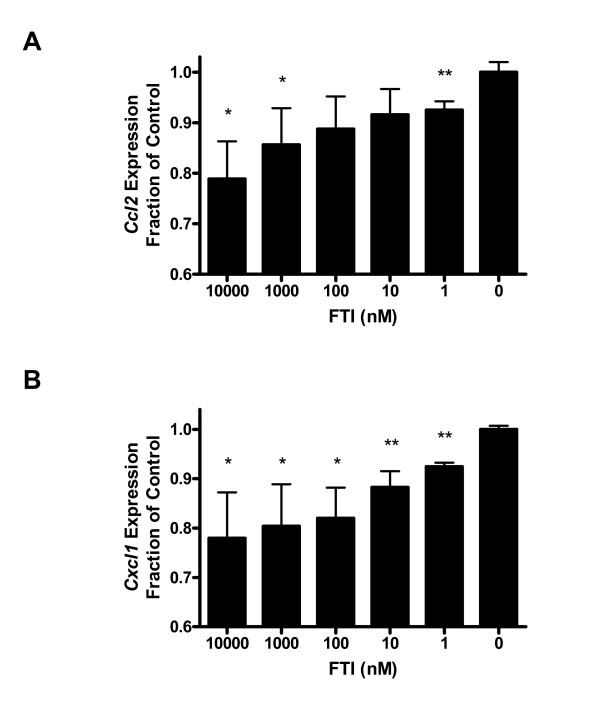
**Reduced expression of pro-inflammatory chemokines in FTI-treated thyrocytes**. **(A) **mRNA expression of the pro-inflammatory chemokine *Ccl2 *in PC Cl3^RP3 ^thyrocytes cultured with indicated concentrations of FTI for 24 hours. **(B) **mRNA expression of the pro-inflammatory chemokine *Cxcl1 *in PC Cl3^RP3 ^thyrocytes cultured with indicated concentrations of FTI for 24 hours. For both chemokines investigated, significant reduction in gene expression is seen, even at nanomolar concentrations of FTI. Data are normalized to *G3pdh *expression within each sample. Error bars represent s.e.m. and are representative of three independent experiments performed in triplicate (n = 3). *p < 0.05, **p < 0.01.

**Figure 3:**
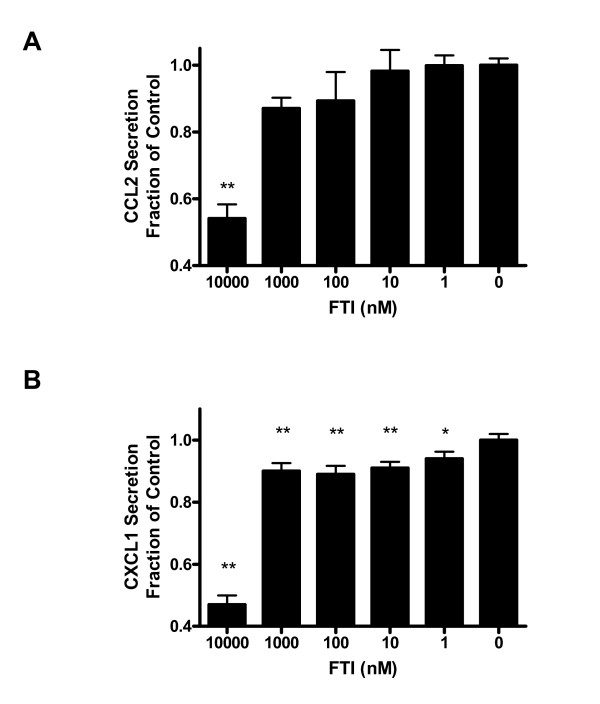
**Reduced pro-inflammatory protein secretion in FTI-treated thyrocytes**. **(A) **Ccl2 protein secretion measured by ELISA from PC Cl3^RP3 ^thyrocytes cultured with indicated concentrations of FTI for 24 hours. **(B) **Cxcl1 protein secretion measured by ELISA from PC Cl3^RP3 ^thyrocytes cultured with indicated concentrations of FTI for 24 hours. Significant reduction of protein secretion compared to control is seen for both chemokines. Error bars represent s.e.m. and are representative of three independent experiments performed in triplicate (n = 3). *p < 0.05, **p < 0.01.

Using *Cxcl1 *and *Ccl2 *as biomarkers for RP3-induced inflammation, we found that that serial dilutions of FTI reduced or abrogated the expression of chemokine mRNA (Figures 2 and 3). Indeed, RT-PCR data shown in Figure 2 revealed a dose-dependent decrease in the gene expression of both *Ccl2 *and *Cxcl1 *after RP3-expressing thyroid cells (PC Cl3^RP3^) were treated with FTI; transcription of *G3pdh *and RP3 itself were unaffected by the same concentrations of FTI, and therefore chemokine transcription was normalized to *G3pdh *(data not shown). This FTI-mediated inhibition of pro-inflammatory gene expression in RP3-expressing cells is dose responsive and significant to nanomolar FTI concentrations.

### Pro-inflammatory protein secretion is inhibited by FTI in RP3-expressing thyrocytes

**Figure 4:**
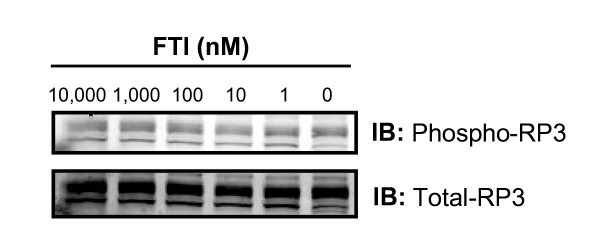
**No effect of FTI on oncogene expression and phosphorylation**. Shown is an immunoblot analysis of RP3 and phospho-RP3 protein expression in PC CL3^RP3 ^thyrocytes cultured with indicated concentrations of FTI for 24 hours. RP3 protein expression and phosphorylation was unchanged at any concentration of FTI. The data are representative of three independent experiments performed in triplicate.

In order to further elucidate the level of FTI inhibition in RP3-expressing cells, we quantified chemokine protein synthesis in the presence and absence of FTI. Several groups have shown that RP3 induces nuclear translocation of NFκB and the subsequent release of multiple inflammatory mediators as well as Class II MHC and co-stimulatory molecules [23,34]. Accordingly, by potentially blocking the oncogene-induced signal transduction pathway, FTI treatment should inhibit pro-inflammatory protein secretion. ELISA data (Figure 3) demonstrates a significant reduction in the secretion of pro-inflammatory mediators CCL2 and CXCL1 in RP3-expressing thyrocytes (PC Cl3^RP3 ^cells). Reductions in protein secretion are evident at nanomolar concentrations of FTI, consistent with mRNA expression data shown in Figure 2. Because inhibition of farnesylation with FTI has the potential to alter intracellular localization of proteins other than RAS, we evaluated the expression of the RP3 oncogene itself in transfected cells after FTI treatment. Data shown in Figure 4 indicate that RP3 protein expression and phosphorylation was unchanged at any concentration of FTI tested. These data provide evidence that FTI is likely acting post-translationally to block RP3-induced signaling and not simply inhibiting expression of RP3 itself, leading to a subsequent reduction in pro-inflammatory mediator expression.

### FTI does not significantly affect apoptosis in RP3-expressing thyrocytes

**Figure 5:**
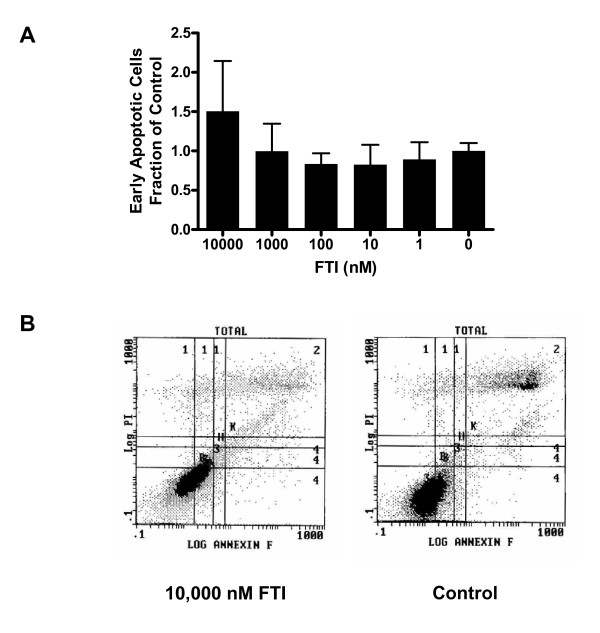
**Apoptosis is unaffected in FTI-treated thyrocytes**. **(A) **Shown are data from FITC-conjugated Annexin V and PI stained PC Cl3^RP3 ^thyrocytes cultured with indicated concentrations of FTI for 24 hours and analyzed by flow cytometry. Cells staining with Annexin V only represent early apoptotic cells, and the percentages of this population at indicated concentrations of FTI are depicted in the histogram (n = 3). There is no significant change in induction of apoptosis at any concentration of FTI. **(B) **Shown are representative dot plots from flow cytometry. Error bars represent s.e.m. and are representative of three independent experiments.

The argument could be made that loss of chemokine gene expression in RP3-expressing cells treated with FTI may be associated with cell death since high concentrations of the agent could be toxic due to direct or indirect effects. To investigate this possibility, PC Cl3^RP3 ^cells co-cultured with increasing concentrations of FTI were stained with Annexin V and propidium iodide (PI) and analyzed by flow cytometry. Cells that stained positive for Annexin V but negative for PI indicated that they were in the early apoptotic stage and thus could be distinguished from live (negative for both markers) and late-apoptotic/dead cells (positive for both markers). Figure 5 demonstrates no significant difference in induction of apoptosis in FTI-treated PC Cl3^RP3 ^cells compared to the same untreated control cells.

## Discussion

The association between cancer and inflammation is well established [33]. However, the mechanisms that govern this association are not well understood. Two notions have been put forth to help explain this phenomenon: one posits that the carcinogenic nature of activated inflammatory cells initiates transforming mutations while another suggests that inflammation is a response to neoplastic transformation and is responsible for tumor progression [35]. Histochemical analyses often used to characterize the actions of inflammatory cells at cancer sites may not provide a complete look into the complexities of how the tumor microenvironment is operating. In recent years, several groups [8,36] have demonstrated activation of the RET/PTC oncogene in the thyroids of humans with autoimmune thyroiditis without thyroid cancer. In such cases, the relationship between cancer and inflammation could instead be interpreted with a view that oncogenic transformation (e.g. oncogene expression) is the basis for the observed inflammation. Along these lines, RET/PTC3 can induce pro-inflammatory activities from thyroid epithelial cells [23,14,13], and thus inhibition of such inflammation may have implications for disease control.

Farnesyltransferase inhibitors are a class of small-molecule agents developed as a novel approach to anti-cancer treatment, designed to target a post-translational modification required for functionality of certain membrane-associated proteins, including RAS. These molecules were targeted based on evidence that many human cancers contain mutations of RAS proteins. Although clinical trials of farnesyltransferase inhibitors demonstrated low toxicity, clinical efficacy was also low in several malignancies. Some scientists have postulated that this may be due to a lack of Ras signaling in later stages of tumor development, while others look to alternate pathways potentially affected by off- or on-target effects of FTI. These failures notwithstanding, more recent data has demonstrated efficacy of FTI as an anti-inflammatory agent [26,27] in both cell- and animal-based models of inflammation. FTI activity has been shown to inhibit the expression of NFκB [26] as well as pro-inflammatory cytokines such as *Ccl2*, *Il6*, and *Ifnβ *[27] induced by carcinogens and inflammatory stimuli.

We have shown here that clinically-relevant nanomolar doses of FTI significantly reduce the expression of pro-inflammatory mediators *Ccl2 *and *Cxcl1*, shown to be two of the chemokines most highly induced by RP3 [23]. We further demonstrate that this reduction is not due to a similarly decreased expression of the oncogene itself; FTI is likely acting post-translationally. We postulate that the effects we have demonstrated with these experiments are due to FTI acting to block RP3 signaling through the RAS pathway, inhibiting NFκB activation, and resulting in decreased expression of pro-inflammatory mediators; however we have not provided any new evidence regarding signaling in the experiments described here.

The current "gold standard" of therapy for autoimmune thyroiditis is lifelong hormone replacement therapy, which treats the symptoms while allowing the disease to run its course. However, failing to treat the underlying cause of autoimmune disease leads to unabated destruction of the affected organ. Indeed, thyroid function is not restored with simple hormone supplementation, and many patients continue to suffer from potentially life-threatening symptoms including obesity, depression, infertility, and gastrointestinal abnormalities due to sub-clinical hypothyroidism. With twelve different doses of synthetic thyroid hormone available, achieving near-exact levels of endogenously-produced thyroid hormone is extremely difficult, and can lead to both sub-clinical hypo- and hyperthyroidism. Importantly, hormone replacement therapy does not stop the progression of differentiated thyroid carcinomas, thought by some to be associated with autoimmune thyroiditis [4]. Implications from the experiments described here may suggest the application of FTI in treating thyroid autoimmune inflammation caused by oncogene signaling. Indeed, the dominant role for aberrant signaling following the expression of the RET/PTC oncoprotein has implicated the Ras and NFκB pathways and helps to explain the production of pro-inflammatory mediators by these transformed epithelial cells [23]. The use of FTI to inhibit RP3 signaling would represent a novel tissue-targeted therapy for thyroid autoimmune disease. Such an approach could provide for the maintenance or recovery of thyroid function before the permanent loss of thyroid hormone ensues following irreversible autoimmune destruction. Future studies may provide a better understanding of the pathways that are shared between autoimmune disease and cancer of the thyroid.

## Competing interests

The author(s) declare that they have no competing interests.

## Authors' contributions

KD carried out all studies and statistical analyses, and drafted the manuscript. BD contributed to the immunoassays. JT performed similar pilot studies and contributed to design of FTI assay. JR conceived of the study, and participated in its design and coordination. All authors read and approved the final manuscript.
